# IL‐6 and IL‐17A degradation by mast cells is mediated by a serglycin:serine protease axis

**DOI:** 10.1002/iid3.95

**Published:** 2015-12-02

**Authors:** Ida Waern, Iulia Karlsson, Gunnar Pejler, Sara Wernersson

**Affiliations:** ^1^ Department of Anatomy, Physiology, and BiochemistrySwedish University of Agricultural SciencesUppsalaSE‐75007Sweden; ^2^ Department of Medical Biochemistry and MicrobiologyUppsala UniversityUppsalaSE‐75123Sweden

**Keywords:** Cytokines, IL‐6, IL‐17, mast cells, proteoglycans, serglycin, serine proteases

## Abstract

Mast cells contain large amounts of fully active proteases that are stored in complex with serglycin proteoglycan in their secretory granules. Upon degranulation, such serglycin:protease complexes are released to the extracellular space and can potentially have an impact on the local inflammatory reaction, either through direct effects of serglycin proteoglycan or through effects mediated by its bound proteases. The objective of this study was to address this scenario by investigating the possibility that serglycin‐associated proteases can regulate levels of pro‐inflammatory cytokines. Indeed, we show here that activated cultured peritoneal mast cells from wild type mice efficiently reduced the levels of exogenously administered IL‐6 and IL‐17A, whereas serglycin‐deficient mast cells lacked this ability. Furthermore, our data suggest that the reduction of IL‐6 and IL‐17A concentrations is due to proteolytic degradation mediated by serglycin‐dependent serine proteases. Moreover, we show that activated mast cells have the capacity to release IL‐6 and that the levels of this cytokine in supernatants were markedly higher in cultures of serglycin‐deficient versus serglycin‐sufficient mast cells, suggesting that serglycin‐dependent serine proteases also participate in the regulation of endogenously produced IL‐6. In summary, although the general consensus is that mast cells have a pathogenic impact on inflammatory settings, this study identifies a role for a mast cell‐derived serglycin:serine protease axis in down‐regulating levels of major inflammatory cytokines. These findings support the notion that mast cells could have a dual role in inflammatory settings, by both being able to secrete pathogenic compounds and being able to regulate their levels after release.

## Introduction

Mast cells are mainly known to be key effector cells in allergic reactions but are also important immune cells in the defense against bacteria and several toxins 1. Mast cells mainly exert their functions through the release of a wide variety of granule‐stored and de novo‐synthesized mediators 1, 2. One of the major granule components is serglycin proteoglycan, which is composed of a core protein to which highly sulfated heparin chains are attached. It has been shown that the negatively charged heparin side chains of serglycin are essential for promoting the storage of positively charged mast cell‐restricted proteases, that is, chymases, tryptases, and carboxypeptidase A3 3. Moreover, serglycin has been shown to be of importance for regulating the enzymatic activities of such proteases 4.

Several previous studies have implicated serglycin and its associated proteases, that is, a “serglycin:protease axis” in regulating inflammatory responses at different levels. For example, it has been shown that serglycin‐deficient mice exhibit age‐related enlargement of lymphoid organs including the spleen, Peyer's patches, and bronchus‐associated lymphoid tissue 5, suggesting a role for serglycin, and possibly its associated compounds, in maintaining homeostasis of leukocyte populations. In further support for a role of a serglycin:protease axis in regulation of inflammation, the knockout of individual serglycin‐dependent proteases has been shown to impact on inflammatory responses in various settings. We recently showed that a murine chymase, mouse mast cell protease 4 (mMCP4), is protective in a house dust mite‐induced asthma model, possibly by regulating the levels of the pro‐inflammatory cytokine interleukin 33 (IL‐33) 6 and, similarly, mMCP4 was shown to be protective in an ovalbumin (OVA) model of allergic airway inflammation 7. It has also been demonstrated that serglycin‐associated mast cell proteases can regulate levels of tumor necrosis factor α (TNF‐α), and various venom‐derived toxins 8, 9, 10, and there are indications that serglycin‐dependent proteases can regulate the levels of IL‐13 11. Interestingly, regulation of both external and endogenously produced IL‐6 and IL‐13 by human mast cells has been shown to involve proteolytic degradation by chymase and cathepsin G 12. In contrast, murine chymase mMCP4 and murine cathepsin G were unable to degrade IL‐13 11, and degradation of IL‐6 was exerted by the tryptase mMCP6 and not by mMCP4 13. Despite these species differences, studies of both human and mouse mast cells support a role for the serglycin:protease axis in the down‐regulation of inflammatory cytokines. It should, however, be noted that the serglycin:protease axis may also have an indirect and opposite effect on cytokine levels, as was suggested by studies showing that rat mast cell granules as well as purified mast cell proteases and histamine can enhance IL‐6 production by activated endothelial cells 14, 15.

IL‐6 is a versatile cytokine that is highly expressed during infection and inflammation. IL‐6 has a broad variety of functions, for example, in regulating proliferation, migration, and differentiation of target cells including T helper 17 cells 16. Appropriate regulation of IL‐6 is crucial since an increased level of IL‐6 has been connected with autoimmune diseases and acute as well as chronic inflammation 17, 18, 19. IL‐17 is a pro‐inflammatory cytokine expressed by a specific type of T lymphocytes, T helper 17 cells but also by several other immune cells, for example, γδ T cells, macrophages, natural killer cells, and natural killer T cells. IL‐17 plays a protective role in the clearance of bacteria and controlling fungal infection 20. However, dysregulation of IL‐17 can result in chronic inflammatory conditions such as psoriasis, rheumatoid arthritis, and multiple sclerosis 21.

In this study, we evaluated the impact of terminally differentiated mast cells on a range of pro‐inflammatory cytokines, and if serglycin‐dependent proteases could account for such activities. Indeed, we show that activated mast cells have the capacity to reduce the levels of both IL‐6 and IL‐17A. Furthermore, we demonstrate that the regulation of these cytokines is dependent on serine proteases that are dependent on serglycin. Taken together, this study supports the notion that mast cells, although mainly regarded as pro‐inflammatory cells, also can function as regulatory cells by degrading inflammatory mediators via a serglycin:serine protease axis.

## Materials and Methods

### Mice

Serglycin^−/−^ 22 and wild‐type (WT) C57BL/6 mice were used in the experiments. Mice were bred and maintained in the National Veterinary Institute (SVA, Uppsala, Sweden). Animal experiments were approved by the local ethical committee.

### Generation and phenotypic characterization of PCMCs

Peritoneal cell‐derived mast cells (PCMCs) were established according to a previously described protocol 23. Briefly, PCMCs were established by culture of peritoneal cells in DMEM plus GlutaMAX (Gibco, Life Technologies, Paisley, UK) supplemented with 10% supernatant of stem cell factor‐transfected Chinese hamster ovary cells (a generous gift from M. Daeron, Pasteur Institute, France), 10% FBS, 60 μg/ml streptomycin, 50 μg/ml penicillin, 100 μM MEM non‐essential amino acids, and 50 μM 2‐mercaptoethanol (Gibco). Medium was changed every fourth to fifth day. Cultures of 4–5 weeks were used for all experiments and the generation of a homogenous (>99% pure) mast cell population was confirmed by Toluidine staining of cytospin slides 11. Previous phenotypic characterizations of PCMCs have demonstrated the presence of large amounts of preformed granule mediators (including mMCP4, ‐5, ‐6, and CPA3) and potent proteolytic activity has been confirmed in supernatants collected from degranulated PCMCs 6, 11, 23. In contrast to WT counterparts, serglycin‐deficient PCMCs did not contain any detectable amounts of mMCP4, ‐5, ‐6, or CPA3, and serglycin‐deficient peritoneal extracts were virtually depleted of trypsin‐, chymotrypsin‐, and CPA‐like activity 11, 22. To confirm the phenotype of WT and serglycin^−/−^ PCMCs, we measured protease activity in supernatants collected 1 h after activation with either 2 μM calcium ionophore A23187 (Sigma–Adrich, St Louis, MO) or with FcϵRI cross‐linking (described in a later section). Chymase‐ and trypsin‐like activities were measured using fluorogenic substrates (Suc‐Ala‐Ala‐Pro‐Phe‐AMC and Boc‐Val‐Pro‐Arg‐AMC) according to the manufacturers instructions (Bachem, Weil am Rhein, Germany). CPA3 activity was measured by the decrease in absorbance at 405 nm using the chromogenic substrate M‐2245 from Bachem (Bubendorf, Switzerland), as previously described 24.

### Cytokine array

The RayBio mouse cytokine antibody array 2 (RayBiotech, Norcross) was used for detecting the levels of added (20 ng/ml) IL‐3, IL‐4, IL‐6, IL‐9, IL‐10, IL‐13, IL‐17A, eotaxin, GM‐CSF, IFN‐γ, and TNF‐α in 1 ml medium supernatant from PCMCs (1 × 10^6^ cells/ml) stimulated with calcium ionophore A23187 at a final concentration of 2 μM (Sigma–Adrich). Recombinant cytokines were from ImmunoTools, Friesoythe, Germany. Samples were collected 4 h after addition of the cytokines. Samples with cytokines in medium alone (no added PCMCs) were used as controls. The spot densities were evaluated using densitometry, as previously described 7. Four hours after adding 2 μM A23187 to PCMC cultures, the cell viability was >91% as determined by trypan blue exclusion.

### IL‐6 and IL‐17A degradation

PCMCs were degranulated via cross‐linking of FcϵRI. IgE anti‐TNP (BD Biosciences Pharmingen, San Diego, CA) was added to 1 × 10^6^ PCMCs at a concentration of 1 μg/ml followed by incubation over night at 37°C. Cells were washed three times and resuspended in DMEM supplemented as above. OVA‐TNP was added to a final concentration of 0.4 µg/ml to 1 × 10^6^ cells/ml. For calcium ionophore‐mediated degranulation, A23187 (Sigma–Aldrich) was added (2 μM final concentration) to 1 × 10^6^ cells/ml. The cell viability was >94% 1 h after activation of PCMCs. In analyses of externally added cytokines, supernatants were collected 1 h after activation and cells were removed by centrifugation. Mouse recombinant IL‐6 and IL‐17A (eBioscience, San Diego, CA) were added in a concentration of 0.5 ng/ml to cell‐free supernatants and levels of remaining cytokines were analyzed after 1, 4, and 24 h. In the analysis of endogenous cytokines, no external cytokines were added and supernatants were collected 1, 4, and 24 h after stimulation. All experiments were performed in triplicates and non‐stimulated cells were used as controls.

### Cytokine analysis

Levels of IL‐6 and IL‐17A were analyzed by enzyme‐linked immunosorbent assay (ELISA) according to the manufacturer's instructions (eBioscience).

### Serine protease inhibitor

PCMCs were degranulated by IgE receptor cross‐linking or by stimulation with calcium ionophore A23187. A serine protease inhibitor, Pefabloc SC (2 mM final concentration; Pentapharm LTD, Basel, Switzerland), was added 30 min prior to the cytokines. Supernatants were collected 1, 4, and 24 h after cytokine addition and analyzed for IL‐6 and IL‐17A by ELISA.

### RNA preparation and real time (RT) PCR analysis

Total RNA from degranulated or control PCMCs were isolated using the NucleoSpin RNA II kit (Merchery‐Nagel, Duren, Germany) and the samples were subjected to reverse transcription using the iScript cDNA synthesis kit (Bio‐Rad). Quantitative PCR was performed in a final volume of 10 µl containing 200 nM primers, iQ SYBR green Supermix (Bio‐Rad), and 1 µl of cDNA. mRNA transcripts were measured on a 7900HT Fast RT‐PCR system and analyzed by SDS 2.3 software (Applied Biosystems, Foster City, CA). Samples were cycled 40 times at 95°C for 30 s, 59°C for 20 s, and 72°C for 20 s, and a melting curve from 60 to 90°C was generated at the end of every run. Primer efficiency was based on a standard curve of dilution 1:1, 1:10, and 1:100 for the primer set. The IL‐6 data were normalized to the cDNA from the housekeeping gene hypoxanthine guanine phosphoribosyl transferase (Hprt) and to the cDNA from IL‐6 in control cells and plotted as mean fold change (+/− SEM).

### Statistical analysis

Statistical significance was calculated with Student's *t*‐test, using the GraphPad Prism 4.0 (GraphPad Software, Inc). All *P*‐values <0.05 were considered to be significant.

## Results

### Reduction of exogenously administered cytokines by activated mast cells

To study the immunoregulatory role of mast cells we tested their ability to reduce the levels of a panel of pro‐inflammatory cytokines. WT or serglycin‐deficient (serglycin^−/−^) mast cells were induced to degranulate by stimulation with the calcium ionophore A23187. After activation, recombinant IL‐3, IL‐4, IL‐6, IL‐9, IL‐10, IL‐13, IL‐17A, eotaxin, GM‐CSF, INF‐γ, and TNF‐α was added to the cell culture medium. After a further incubation period of 4 h, samples were taken and were analyzed for content of residual cytokines by using a cytokine antibody array approach.

**Figure 1:**
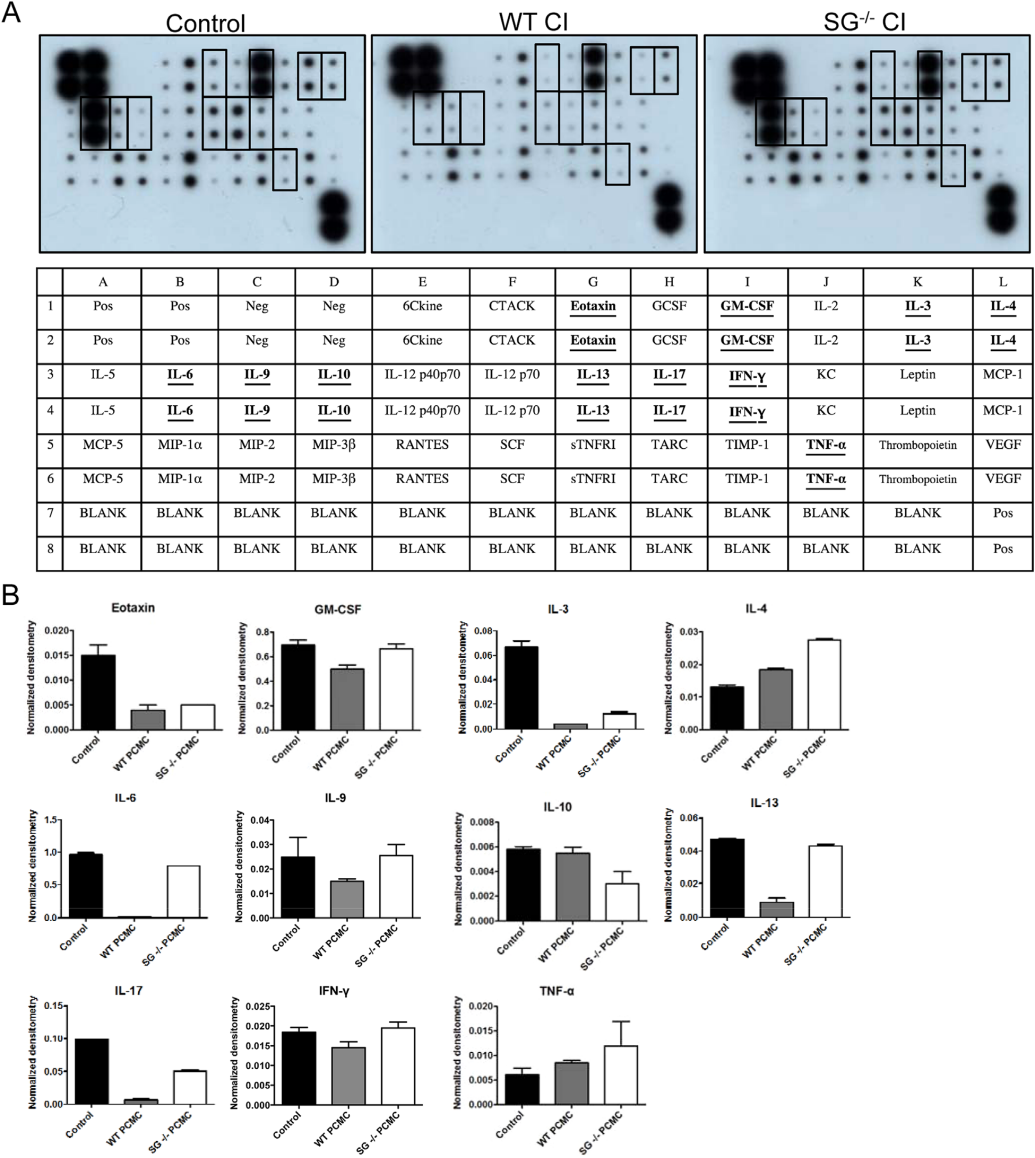
Serglycin‐dependent regulation of exogenously administered cytokines. (A) Peritoneal cell‐derived mast cells (PCMCs) from WT and serglycin^−/−^ mice were stimulated with calcium ionophore A23187 (CI) in the presence of IL‐3, IL‐4, IL‐6, IL‐9, IL‐10, IL‐13, IL‐17A, eotaxin, GM‐CSF, IFN‐γ, and TNF‐α, all at 20 ng/ml. Samples were taken at 4 h post‐activation. Medium alone with addition of the cytokines was used as control. Cytokine levels in the supernatants were detected with a mouse cytokine antibody array. Layout of the mouse cytokine antibody array. (B) Cytokine array dots were quantified by densitometry. The duplicate values for each cytokine were normalized to positive control dots, represented on each membrane. Data are presented as means ± SEM (*n* = 2).

As visualized by the antibody array filter (Fig. 1A) and by the densitometry analysis (Fig. 1B), activated WT mast cells had the capacity to profoundly reduce the levels of IL‐3, IL‐6, IL‐13, IL‐17A, and eotaxin. In contrast, the levels of IL‐4, IL‐9, IL‐10, GM‐CSF, IFN‐γ, and TNF‐α were either unaffected or only affected to a minor extent by the activated mast cells. The reduction of IL‐3 and eotaxin did not differ between WT and serglycin^−/−^ mast cells, suggesting that these compounds are regulated by mast cells through serglycin‐independent mechanisms. In contrast, it was clear that the effects of activated mast cells on exogenous IL‐6, IL‐13, and IL‐17A were blunted in cultures of serglycin^−/−^ mast cells. Hence, these findings suggest that mast cells can regulate exogenous IL‐6, IL‐13, and IL‐17A and that this ability is dependent on a serglycin proteoglycan‐mediated pathway.

### Degradation of IL‐6 and IL‐17A by mast cells is dependent on a serglycin:serine protease pathway

**Figure 2:**
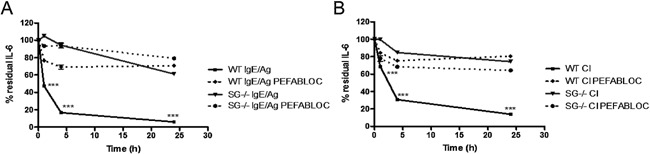
IL‐6 regulation by mast cells is mediated by serglycin‐dependent serine proteases. (A and B) Mast cell degranulation was induced by IgE‐receptor crosslinking (IgE/Ag) or by calcium ionophore A23187 (CI). IL‐6 was added to the cell‐free supernatant containing the degranulate in the presence of a serine protease inhibitor (Pefabloc SC; 2 mM final concentration). Medium alone with addition of IL‐6 was used as control. The levels of IL‐6 in supernatants taken after 1 h, 4 h, and 24 h were measured by ELISA. Data were calculated as percent residual IL‐6 compared to control. Data are expressed as means ± SEM. ***P < 0.001 for degranulated WT sample versus degranulated serglycin^−/−^ sample, and WT sample with inhibitor versus no inhibitor.

In order to confirm the findings from the cytokine array analysis by a quantitative and kinetic approach we used IL‐6 and IL‐17A ELISA. For these analyses, we activated mast cells by either calcium ionophore stimulation or by IgE receptor (FcϵRI) cross‐linking, the latter representing a physiologically relevant mode of mast cell activation. As depicted in Figure 2, the levels of IL‐6 in the supernatant of WT mast cells, after stimulation with either FcϵRI cross‐linking or calcium ionophore, was markedly reduced already after 1 h. After 4 and 24 h the reduction was even more pronounced. In contrast, there was only a minor effect on IL‐6 levels in supernatants from activated serglycin^−/−^ mast cells (Fig. 2A and B). This suggests that a serglycin proteoglycan‐dependent mechanism has a major regulatory impact on the levels of IL‐6.

**Figure 3:**
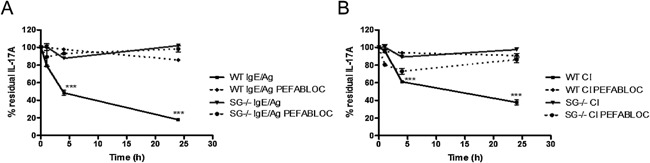
Mast cell serine proteases degrade IL‐17A. (A and B) WT and serglycin^−/−^ PCMCs were degranulated using either IgE‐anti‐TNP and OVA‐TNP (IgE/Ag) or by calcium ionophore A23187 (CI). IL‐17A was added to the cell‐free supernatant, containing the degranulate, in the presence or absence of a serine protease inhibitor (Pefabloc SC). The levels of IL‐17A in the supernatant after 1, 4, and 24 h were measured with ELISA. Data were calculated as percent residual IL‐17A compared to control. Data are expressed as means ± SEM. ***P < 0.001 in degranulated WT sample versus degranulated serglycin^−/−^ sample, and WT sample with inhibitor versus no inhibitor.

Similar to the effects on IL‐6, mast cell activation with either calcium ionophore or IgE receptor cross‐linking also caused a rapid reduction of IL‐17A levels in the culture supernatants (Fig. 3A and B). Moreover, the reduction of IL‐17A was highly dependent on a serglycin‐mediated mechanism, that is, virtually no reduction of exogenously administered IL‐17A was seen in supernatants of serglycin^−/−^ mast cells (Fig. 3), which is in concordance with the data on IL‐6.

To approach the mechanism behind the serglycin‐dependent regulation of IL‐6 and IL‐17A protein levels, we considered the possibility that the reduction of the cytokine concentrations was mediated by serglycin‐associated serine proteases. The rationale behind this was that several different mast cell serine proteases are known to depend on serglycin for storage and there are also previous studies implicating such serine proteases in the regulation of various cytokines by proteolytic degradation 6, 11, 12, 25. To test this hypothesis, we analyzed whether a serine protease inhibitor, Pefabloc SC, could block reduction of cytokine concentrations. Indeed, the addition of Pefabloc SC caused an essentially complete blockade of the reduction of both IL‐6 and IL‐17A, strongly suggesting that the regulation of these cytokines by mast cells is due to proteolytic degradation catalyzed by serglycin‐associated serine proteases. Moreover, we confirmed the presence of chymase‐ and tryptase‐like activity in supernatants from activated WT PCMCs, but not serglycin^−/−^ PCMCs (Table 1). Taken together, these findings suggest that the reduction of exogenously administered IL‐6 and IL‐17A by mast cell releasates is strongly dependent on proteolytic activity executed by a serglycin:serine protease axis.

**Table 1:** Protease activities in mast cell releasates^1^

	IgE/Ag			CI		
	WT	Serglycin^−/−^	*P*‐value	WT	Serglycin^−/−^	*P*‐value
Chymase‐like^2^	32.2 ± 1.54	0.18 ± 0.43	<0.001	204 ± 12.4	16.7 ± 1.38	<0.001
Tryptase‐like^2^	301 ± 6.67	8.83 ± 1.19	<0.001	2360 ± 48.8	31.5 ± 1.44	<0.001
CPA‐like^3^	−1.42 ± 0.07	−0.09 ± 0.08	<0.001	−5.25 ± 0.02	0.24 ± 0.01	<0.001

1 WT or serglycin^−/−^ PCMCs were activated with IgE‐receptor crosslinking (IgE/Ag) or calcium ionophore A23187 (CI). Supernatants were collected after 1 h and analyzed for chymase‐, tryptase‐, and CPA‐like activities by adding fluorogenic or chromogenic substrates. Experiments were performed in triplicates.

2 Activities are given as mean ± SEM of raw fluorescence units (RFU)/(min × 10^6^ cells).

3 Activities are given as mean ± SEM of ΔmOD/(min × 10^6^ cells). Note that hydrolysis of the CPA substrate results in a decreased absorbance.

### A serglycin:serine protease axis regulates IL‐6 that is endogenously produced by mast **cells**

**Figure 4:**
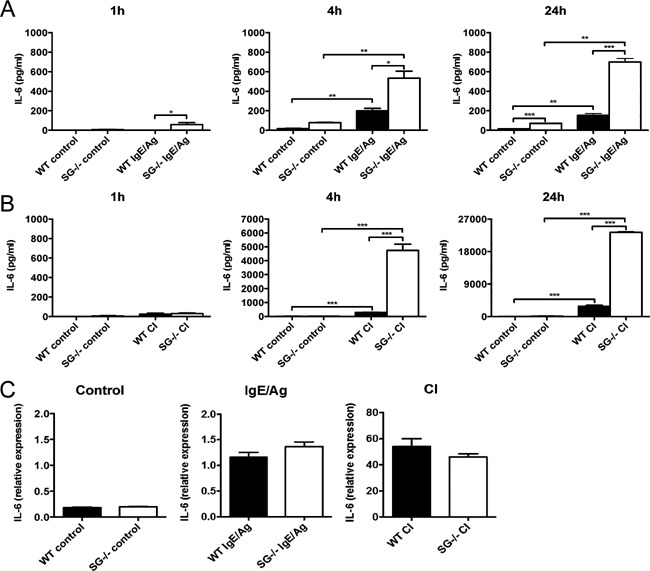
Degradation of endogenous IL‐6 is abrogated in serglycin^−/−^ mast cell cultures. (A and B) WT and serglycin^−/−^ PCMCs were degranulated by IgE receptor crosslinking (IgE/Ag) or by calcium ionophore A23187 (CI). Non‐stimulated cells were used as controls. The levels of endogenously produced IL‐6, 1, 4, and 24 h after stimulation, were analyzed by ELISA. Data are from representative experiments and are expressed as means ± SEM. *P < 0.05; **P < 0.01; ***P < 0.001. (C) IL‐6 mRNA levels in control mast cells (left panel), mast cells stimulated by IgE receptor crosslinking (IgE/Ag; middle panel) and mast cells stimulated by calcium ionophore (CI; right panel). Mast cells from either WT or serglycin^−/−^ (SG^−/−^) mice were analyzed. Results are expressed as mRNA expression relative to the housekeeping gene (*Hprt*) and relative to controls. Note that the levels of IL‐6 gene expression were not significantly different between WT and serglycin^−/−^ mast cells.

Mast cells have been shown to produce a number of cytokines upon activation, for example, IL‐6, IL‐13, and TNF‐α 1. To determine whether a serglycin:serine protease axis also could regulate the levels of cytokines endogenously produced by mast cells, mast cells were stimulated with either IgE receptor cross‐linking or calcium ionophore, followed by measurements of released IL‐6 and IL‐17A. As seen in Figure 4A and B, respectively, robust secretion of IL‐6 was seen in response to both IgE receptor cross‐linking and calcium ionophore stimulation after 4 and 24 h. In contrast, IL‐17A was not detected in supernatants taken from stimulated mast cells (data not shown). It was also noted that the levels of secreted IL‐6 were profoundly higher in supernatants from cultures of activated serglycin^−/−^ as opposed to WT mast cells (Fig. 4A and B). Hence, these findings suggest that, similar to the regulation of exogenously administered IL‐6, endogenous production of IL‐6 by mast cells is the subject to regulation by a serglycin‐dependent mechanism.

The elevated levels of IL‐6 seen in cultures of serglycin^−/−^ mast cells could potentially be the result of either defective degradation or increased expression. To evaluate the latter possibility, RNA was extracted from non‐stimulated control cells and from mast cells that had been activated with either IgE receptor cross‐linking or with calcium ionophore, followed by quantification of IL‐6 mRNA by quantitative real time RT‐PCR. As shown in Figure 4C, there was no significant difference in IL‐6 mRNA expression between WT and serglycin^−/−^ mast cells, neither when comparing non‐stimulated nor activated cells (Fig. 4C). Hence, these data suggest that the dramatic difference in levels of endogenous IL‐6 protein in WT versus serglycin^−/−^ mast cells is due to effects at the post‐translational level. Clearly, this is in agreement with a proposed mechanism of IL‐6 regulation where the elevated levels of IL‐6 seen in cultures of serglycin^−/−^ mast cells is explained by defective degradation due to the absence of a serglycin:serine protease axis.

## Discussion

Mast cells contain secretory granules packed with pre‐formed mediators, including large amounts of fully active proteases complex‐bound to serglycin proteoglycans. Upon degranulation, large amounts of these compounds are thus released and may have a profound influence on the surroundings. Potentially, such effects could be either beneficial or harmful, depending on the setting 2. For example, the murine chymase mMCP4 protects against allergic airway inflammation 6, 7, neuroinflammatory conditions 25, and renal fibrosis 26 but has in other settings been shown to be detrimental 27, 28, 29, 30.

As regards the mechanism by which proteases released by mast cells influence an inflammatory reaction, one possible scenario is that such proteases may act on cytokines found in the extracellular milieu. Potentially, the action of mast cell‐released proteases could either lead to proteolytic activation of cytokines, or to cytokine degradation and destroyed biological activity. In accordance with the former possibility, there are reports suggesting that mast cell serine proteases can proteolytically activate certain cytokines 31, 32, 33 and chemokines 34, 35. Conversely, protease‐dependent degradation of cytokines by human mast cells has previously been demonstrated 6, 12. In line with the latter scenario, the present report shows that fully mature peritoneal mast cells have the capacity to regulate levels of IL‐3, IL‐6, IL‐13, IL‐17A, and eotaxin. Interestingly, although all of these cytokines were profoundly affected by peritoneal mast cells, other cytokines, for example, GM‐CSF, appeared to be completely resistant to mast cell‐dependent regulation. Hence, these data suggest that mast cells specifically participate in the regulation of certain cytokines, whereas others may be regulated by mast cell‐independent control mechanisms. Hence, the latter finding is in line with previous studies on human mast cells suggesting that certain cytokines and chemokines, including GM‐CSF, are more resistant to degradation by mast cell‐dependent pathways 12. It should, however, be noted that we cannot exclude the possibility that some of the cytokines that appeared to be resistant to mast cell‐dependent regulation, as detected by the dot blot, could still be subject to a limited proteolysis affecting the cytokine activity without reducing the immunoreactivity.

Another interesting observation in this study was that the regulation of IL‐6, IL‐13, and IL‐17A was abrogated when mast cells lacked serglycin proteoglycan. Hence, these findings identify a novel role for serglycin‐dependent mechanisms in regulating IL‐6 and IL‐17A, and are in agreement with a previous study in which IL‐13 was shown to be regulated by a serglycin‐dependent mechanism 11. To our knowledge, this is the first demonstration of a direct regulation of IL‐17A by mast cell granule components. Although degradation of IL‐6 by several types of mast cell proteases was reported in previous work 12, 13, the total dependency on serglycin proteoglycan demonstrated in the present study confirms that serglycin‐dependent mechanisms are crucial for mast cell‐mediated IL‐6 regulation. However, further investigations are needed to establish whether serglycin is required for limiting the in vivo levels of IL‐6 and IL‐17A during mast cell‐mediated inflammatory conditions.

Our data show that the mast cell‐mediated reduction of IL‐6 and IL‐17A was abrogated in the presence of a serine protease inhibitor. Accordingly, this finding indicates a role for any of the serine proteases that are known to be stored in a serglycin‐dependent manner. These include the mast cell‐restricted serine proteases of chymase (mMCP4, ‐5) and tryptase (mMCP6) type, but there are also indications that additional, unidentified serine proteases can be stored in complex with serglycin and be of biological significance in regulating cytokine levels 11. We are at present not able to identify which of the various serglycin‐dependent serine proteases that is mainly responsible for the degradation of IL‐6 and IL‐17A by peritoneal mast cells. One possible candidate to carry out this function could be chymase, which was shown to be responsible for IL‐6 degradation mediated by human mast cells 12. On the other hand, studies on murine mast cells did not confirm this finding 36. Instead, it was shown that the tryptase mMCP6 (and not the chymase mMCP4) could cleave IL‐6, and that abrogation of this pathway in dipeptidyl peptidase I‐deficient mice could possibly explain their elevated levels of IL‐6 and improved survival in a model of septic peritonitis 13. Hence, it is most likely that mMCP6 participates in the serglycin‐dependent regulation of IL‐6 by peritoneal mast cells. However, it is worth noting the possibility of redundancy among the serglycin‐dependent proteases, that is, the distinct serglycin‐dependent proteases can compensate for each other in the proteolysis of individual substrate proteins. Moreover, there is also the likely possibility that the various mast cell proteases may act in concert by cleaving at different sites of a given substrate. According to such a scenario, the absence of the entire serglycin:protease axis (as imposed by serglycin deficiency) may have a much more dramatic effect on the cleavage of certain compounds than if merely individual proteases are inhibited (see ref. 37 for a discussion). Taken together, we may thus envisage that the serglycin:protease axis may have a profound impact on the extracellular regulation of a range of proteinaceous compounds of importance for regulating the inflammatory response. However, it is important to emphasize that the contribution from individual serglycin‐associated proteases to such functions may vary extensively dependent on the nature of the substrate, specific protease expression profile of a given mast cell population, timing during the inflammatory response and species differences.

The present study also adds to our knowledge of the cytokine repertoire of mast cells. The capacity of mast cells to produce IL‐6 is well established 1, 38 and it has also been shown that mast cell‐derived IL‐6 plays a critical role in various pathological processes, including a protective role in sepsis 39. Importantly, the present study was carried out using fully differentiated peritoneal cell‐derived mast cells (PCMCs) 23, whereas various less mature mast cell populations have often been used in previous studies in which the production of cytokines by murine mast cells has been studied 40. We show that activation of PCMCs with either IgE receptor crosslinking or calcium ionophore stimulated IL‐6 release from PCMCs, thus verifying that secretion of IL‐6 is a property of fully mature murine mast cells. Interestingly, the increased levels of IL‐6 seen in cultures of activated serglycin‐deficient mast cells suggests that levels of endogenously produced IL‐6 is limited by a serglycin‐dependent mechanisms in WT mast cells. This finding is also in line with previous work demonstrating that endogenously produced cytokines can be regulated by mast cell proteases 12. In contrast to IL‐6, we could not detect endogenous IL‐17A production after mast cell activation with IgE/Ag or calcium ionophore A23187 (data not shown), thus being in slight discrepancy with previous studies showing that mast cells can be a source of IL‐17 during inflammatory settings 41, 42. However, it cannot be excluded that activation regimes other than the ones employed in this study are necessary in order to induce IL‐17 production by mast cells.

In summary, we here demonstrate for the first time the direct regulation of IL‐17A by mast cells. Moreover, we identify a role for serglycin‐dependent serine proteases in mast cell‐mediated regulation of IL‐17A and IL‐6. Collectively, our findings support a major role for a serglycin:serine protease axis in regulating inflammatory processes through proteolytic degradation of inflammatory cytokines.

## Author Contributions

IW and IK performed and analyzed the experiments. SW and IW designed the study. IW, SW, and GP wrote the paper.

## Conflict of Interest

The authors declare that they have no conflict of interests.
